# Efficacy of warming systems in mountain rescue: an experimental manikin study

**DOI:** 10.1007/s00484-020-02008-6

**Published:** 2020-09-01

**Authors:** Paweł Podsiadło, Ewa Zender-Świercz, Giacomo Strapazzon, Sylweriusz Kosiński, Marek Telejko, Tomasz Darocha, Hermann Brugger

**Affiliations:** 1 Department of Emergency Medicine, Jan Kochanowski University, ul. IX Wieków Kielc 19a, 25-516 Kielce, Poland; 2 Department of Building Physics and Renewable Energy, Faculty of Environmental, Geomatic and Energy Engineering, Kielce University of Technology, Kielce, Poland; 3 Institute of Mountain Emergency Medicine, Eurac Research, Bolzano, Italy; 4 Faculty of Health Sciences, Jagiellonian University Medical College, Krakow, Poland; 5 Faculty of Civil Engineering and Architecture, Kielce University of Technology, Kielce, Poland; 6 Department of Anesthesiology and Intensive Care, Medical University of Silesia, Katowice, Poland

**Keywords:** Hypothermia, Rewarming, Mountain rescue, Thermal manikin, Wind chill index, Cold exposure

## Abstract

Mountain accident casualties are often exposed to cold and windy weather. This may induce post-traumatic hypothermia which increases mortality. The aim of this study was to assess the ability of warming systems to compensate for the victim’s estimated heat loss in a simulated mountain rescue operation. We used thermal manikins and developed a thermodynamic model of a virtual patient. Manikins were placed on a mountain rescue stretcher and exposed to wind chill indices of 0 °C and − 20 °C in a climatic chamber. We calculated the heat balance for two simulated clinical scenarios with both a shivering and non-shivering victim and measured the heat gain from gel, electrical, and chemical warming systems for 3.5 h. The heat balance in the simulated shivering patient was positive. In the non-shivering patient, we found a negative heat balance for both simulated weather conditions (− 429.53 kJ at 0 °C and − 1469.78 kJ at − 20 °C). Each warming system delivered about 300 kJ. The efficacy of the gel and electrical systems was higher within the first hour than later (*p* < 0.001). We conclude that none of the tested warming systems is able to compensate for heat loss in a simulated model of a non-shivering patient whose physiological heat production is impaired during a prolonged mountain evacuation. Additional thermal insulation seems to be required in these settings.

**Electronic supplementary material:**

The online version of this article (10.1007/s00484-020-02008-6) contains supplementary material, which is available to authorized users.

## Introduction

Hypothermia is commonly encountered in mountain rescue operations. Casualties are usually immobilized due to injury and exposed to cold, wind, and humidity (McLennan and Ungersma 1983; Guly 1996; Smith 2006). Exhaustion and energy depletion are frequent, even among uninjured mountaineers, and may cause hypothermia (Hearns 2003). Metabolic heat production is often reduced, while shivering may be attenuated due to severe injury or drug administration, or if one’s core temperature drops below 32 °C (Peng and Bongard 1999; Langhelle et al. 2012). In trauma victims, any drop in core temperature is an independent risk factor for mortality (Shafi et al. 2005). Patients with post-traumatic hypothermia have a higher blood product requirement and a greater risk of multi-organ failure (Martin et al. 2005; Klauke et al. 2016). Mountain rescue operations are usually of longer duration than in urban/suburban environments, even with air support, and may last for several hours if a helicopter cannot be activated due to bad weather (Rauch et al. 2018). The insulation of casualties exposed to a harsh environment by bystanders or first responders is considered a basic measure in order to prevent hypothermia (Paal et al. 2016). Mountain rescue casualty bags are commonly used by rescue teams. Their ability to decrease the victim’s heat loss has been confirmed in both human and manikin studies (Grant et al. 2002; Press et al. 2017). However, clinical trials have shown the substantial role of active external rewarming in order to prevent post-traumatic hypothermia (Lundgren et al. 2011; Langhelle et al. 2012). Dutta et al. compared the impact of different combinations of wrap systems with heating pads on heat balance in humans. However, this study involved normothermic healthy volunteers, and cold exposure lasted only 1 h (Dutta et al. 2019).

Although further heat loss should be avoided during the transport of a hypothermic patient, to the best of our knowledge, the heat balance of a patient lying on a mountain rescue stretcher and the effectiveness of heating measures employed have not yet been investigated. The aim of this study was to assess whether commonly used rewarming systems are able to compensate for the estimated heat loss in various weather and clinical settings during a simulated mountain rescue operation by the use of a thermal manikin on a stretcher.

## Methods

### Study design

This was an experimental manikin study. The thermodynamic model of a virtual patient was developed on the basis of the measurements of the manikin’s heat loss and physiological parameters adopted from human studies on accidental hypothermia. We then calculated the heat balance of this model. Finally, we measured the heat gain from the respective warming systems.

### Assessment of heat loss using the thermal manikin

Manikin 1 was an advanced human body phantom (PT Teknik, Espergærde, Denmark) which measures total heat loss from its entire surface, including convection, radiation, evaporation, and conduction. Its total mass was about 20 kg. Since Manikin 1 had no “core” but only a “shell,” all the energy to warm its surface was emitted into the environment. Thus, no heat storage existed. The device-specific software allowed one to read the surface temperature, the energy supplied, the heat flux for each segment, and the total heat flux. Thus, the total heat loss through the skin over a specific time period could be calculated (Psikuta et al. 2016). As respiratory heat loss (RHL) is not taken into account by the software algorithm, it was calculated separately. Since in this study the ambient air temperature differed significantly from room temperature, we assumed RHL as ~ 20% of metabolic heat production, according to the data from human studies that have assessed thermal balance in a cold environment (Lloyd 1975; Ingenito et al. 1986; Cain et al. 1990).

The intensity of heat flux depends on the temperature gradient between the skin (T_skin_) and the environment (T_ambient_). Manikin 1 could be set to simulate an injured victim with a lower-than-normal skin temperature by using the control mode of stable skin temperature. In this setting, energy delivery is adjusted by the software to maintain the preset T_skin_. The target mean T_skin_ was derived from studies with human participants exposed to a cold environment (Grissom et al. 2008; Thomassen et al. 2011; Oliver et al. 2016). Based on this data, we concluded that we should set the trunk temperature at 25–26 °C and the mean T_skin_ at 23–24 °C. In order to mimic T_skin_ distribution in hypothermic humans, we set the temperature of the distal limbs 8 °C lower than that of the torso (Goheen et al. 1997). Specifically, we set the temperatures as follows: head 27 °C; torso 26 °C; thighs and arms 24 °C; calves and forearms 20 °C; and feet and hands 18 °C. The mean T_skin_, calculated according to Layton et al. (Layton et al. 1983), was 23.4 °C in our study protocol.

The heat loss of Manikin 1 was calculated using the device-specific software (Byteline, ver. 3.4.16).

### Determination of metabolic heat production

We analyzed two clinical scenarios, namely, (1) a shivering patient and (2) a non-shivering patient. These two scenarios characterize the most frequent clinical situations in a prehospital setting, i.e., an uninjured slightly hypothermic shivering patient and an injured hypothermic patient whose shivering has been attenuated due to drug administration or due to the severity of the hypothermia itself.

We derived the metabolic heat production value (*M*) from experimental studies with human participants, identified through a PubMed search. Participants were exposed to a cold environment, where *M* was measured by oxygen consumption. Meperidine was administered in some of these subjects in order to attenuate shivering, allowing one to calculate the mean value of *M* (W/m^2^) for shivering and non-shivering subjects separately. We calculated an *M* value for a shivering victim (M_shiv_) from 11 studies with a total of 77 participants (Giesbrecht et al. 1994; Hultzer et al. 2005; Grissom et al. 2008; Pretorius et al. 2008; Lundgren et al. 2009; Thomassen et al. 2011; Sran et al. 2014; Kumar et al. 2015; Henriksson et al. 2015; Kulkarni et al. 2019; Hurrie et al. 2020). An *M* value for a non-shivering patient (M_non-shiv_) (e.g., a patient treated with opioids) was calculated from six studies with a total of 37 subjects (Giesbrecht et al. 2005; Hultzer et al. 2005; Pretorius et al. 2006; Lundgren et al. 2009; Kulkarni et al. 2019; Hurrie et al. 2020)—see Supplementary file.

### Determination of the total heat balance

The thermal balance of the human body depends on its heat production, loss, and storage. Fanger’s equation (Fanger 1970; Gagge and Gonzalez 1996) adapted to a non-moving subject can be simplified as follows:

1 $$ \mathrm{M}\ \left(\mathrm{W}/{\mathrm{m}}^2\right)=\mathrm{E}+\mathrm{R}+\mathrm{C}+\mathrm{K}+\mathrm{R}\mathrm{HL}+\mathrm{S} $$

where *M* denotes the metabolic heat production, *E* the evaporative heat loss, *R* the heat loss by radiation, *C* the convective heat loss, *K* the heat loss by conduction, *RHL* the respiratory heat loss, and *S* the heat stored in the body (an *S* value of > 0 means heat gain; < 0 means heat loss).

The heat storage (W/m^2^) of the virtual patient was calculated using Eq. 1 for both physiological scenarios: a high metabolic rate associated with shivering (S_shiv_) and low metabolic heat production in a non-shivering victim (S_non-shiv_). We replaced *E*, *R*, *C*, and *K* with *HF*, which is the total heat flux from Manikin 1:

2 $$ {\mathrm{S}}_{\mathrm{shiv}}\ \left(\frac{\mathrm{W}}{{\mathrm{m}}^2}\right)={\mathrm{M}}_{\mathrm{shiv}}\hbox{--} \mathrm{RHL}-\mathrm{HF}\kern0.75em \mathrm{and}\kern0.75em {\mathrm{S}}_{\mathrm{non}-\mathrm{shiv}}\ \left(\mathrm{W}/{\mathrm{m}}^2\right)={\mathrm{M}}_{\mathrm{non}-\mathrm{shiv}}\hbox{--} \mathrm{RHL}\hbox{--} \mathrm{HF} $$

Since we assumed RHL as 0.2 M, we could transform Eq. 2 into:

3 $$ {\mathrm{S}}_{\mathrm{shiv}}\ \left(\frac{\mathrm{W}}{{\mathrm{m}}^2}\right)=0.8\ {\mathrm{M}}_{\mathrm{shiv}}\hbox{--} \mathrm{HF}\kern1em \mathrm{and}\kern1.5em \mathrm{Snon}-\mathrm{shiv}\ \left(\mathrm{W}/{\mathrm{m}}^2\right)=0.8\ {\mathrm{M}}_{\mathrm{non}-\mathrm{shiv}}-\mathrm{HF} $$

The total heat balance (THB) during evacuation (i.e., 3.5 h), expressed in joules (J), was calculated by multiplying heat storage (W/m^2^), body surface area (m^2^), and time (sec) for the shivering and non-shivering scenarios (THB_shiv_ and THB_non-shiv_):

4 $$ {\displaystyle \begin{array}{l}{\mathrm{THB}}_{\mathrm{shiv}}\ \left(\mathrm{J}\right)={\mathrm{S}}_{\mathrm{shiv}\times }\ \mathrm{body}\ \mathrm{surface}\times \mathrm{time}\\ {}\mathrm{and}\\ {}{\mathrm{THB}}_{\mathrm{non}-\mathrm{shiv}}\ \left(\mathrm{J}\right)={\mathrm{S}}_{\mathrm{non}-\mathrm{shiv}}\times \mathrm{body}\ \mathrm{surface}\times \mathrm{time}\end{array}} $$

Specifically, a THB value of < 0 means a decrease in body temperature; conversely an increase in body temperature is shown by a THB value of > 0. A patient body surface area of 1.8 m^2^ was assumed.

### Assessment of heat gain from warming systems

While Manikin 1 is designed to assess heat loss, it cannot assess heat gain as there is no possibility of cooling its surface from the inside. Therefore, the surface can quickly achieve the temperature of a warming pad while no heat flow is being detected. Hence, we designed and built a water manikin (Manikin 2) to measure the amount of heat gained from warming pads. This is a torso-like copper container, painted matt black, filled with 43.3 L of water, with a total mass of 54.77 kg. Manikin 2 was equipped with a precision thermometer-temperature logger (Termio 2, Termoprodukt, Bielawa, Poland) with a probe placed in the geometrical center, along with a water heater and a stirrer. We assessed its emissivity according to Brauer et al. (Brauer et al. 2002). The measured emissivity of Manikin 2 was 0.99 which is close to the emissivity of the human skin (Sanchez-Marin et al. 2009). The average heat flux from Manikin 2 was calculated in a pre-trial test in both weather scenarios and compared with Manikin 1. We observed a difference ranging from 2 to 9%, depending on the ambient temperature.

Rescue services use different types of warming systems, including those which are based on gel (sodium acetate) crystallization, electrical power, or chemical reactions based on iron/carbon powder oxygenation (Hamilton and Paton 1996; Lundgren et al. 2011; Podsiadło et al. 2017). We tested three warming systems based on the three aforementioned methods of heat production, namely:

1. A gel heat pack (ABC-N System. Poland)—three panels, each of which is 45 × 23 cm with a total surface area of 0.32 m^2^ and a total mass of 4.33 kg
2. An electrical blanket (Uniqueresc, Geratherm, Germany)—dimensions of 47 × 90 cm, with a surface area of 0.42 m^2^ and a total mass of 1.7 kg, including an NL2024ED battery (Inspired Energy, USA)
3. A chemical blanket (Ready Heat, Tech Trade, USA)—dimensions of 86 × 122 cm, with a surface area of warming elements of 0.18 m^2^, a total surface area of 1.05 m^2^, and a total mass of 0.794 kg

The temperature change of Manikin 2 served to calculate the total gained or lost heat (Q):

5 $$ \mathrm{Q}\ \left(\mathrm{kJ}\right)=\mathrm{mass}\times \mathrm{specific}\ \mathrm{heat}\times \mathrm{temperature}\ \mathrm{change} $$

We calculated the heat balance of Manikin 2 with the tested warming systems, as well as without a warming system, according to Eq. 5. The difference between the heat balance with (Q_warm_) and without (Q_base_) a warming pad was used to calculate heat gain (HG):

6 $$ \mathrm{HG}\ \left(\mathrm{kJ}\right)={\mathrm{Q}}_{\mathrm{warm}}-{\mathrm{Q}}_{\mathrm{base}} $$

### Study protocol

The study was conducted under controlled and standardized environmental conditions in a climatic chamber (4 × 1.8 × 2.3 m) at 270 m above sea level, with a relative humidity of 40%, and consisted of a sequence of two simulations (A, B) with different wind chill indices. The protocol aimed to simulate an exemplary 3.5-h evacuation of a casualty lying on a mountain rescue stretcher in two different conditions, namely, moderate (A) and harsh (B) weather.

The ambient temperatures (degree Celsius) and wind speeds (m/sec) in the two weather conditions were:

- A – Wind chill index 0 °C (temperature 2 °C, wind speed 2 m/s)
- B – Wind chill index − 20 °C (temperature − 10 °C, wind speed 9 m/s)

We calculated the wind chill index according to that set by the American National Oceanic and Atmospheric Administration (https://www.weather.gov/epz/wxcalc_windchill) and produced wind with two electrical fans (Minneapolis Blower Door model 4.1, The Energy Conservatory Inc., USA).

Initially, we measured the insulation properties of clothing and the stretcher cover (expressed in clo units, 1 clo = 0.155 m2 KW^−1^) with the Manikin 1 software, in accordance with EN ISO 15831:2004. For this purpose, we dressed the manikin in clothes suitable for mountaineering (i.e., pants made of Cordura® with an insulating layer of polyester (fleece), a long-sleeved shirt of polyester, a Polartec® pullover, a soft-shell jacket, Polartec® mitts, mountain boots, and a woolen cap). Then, the manikin was placed on a Lecco mountain stretcher (Kong, Monte Marenzo, Italy) and wrapped up with the integrated cover. This single-layer cover is made from a windproof and waterproof fabric, although it does not have any additional insulation.

**Fig. 1:**
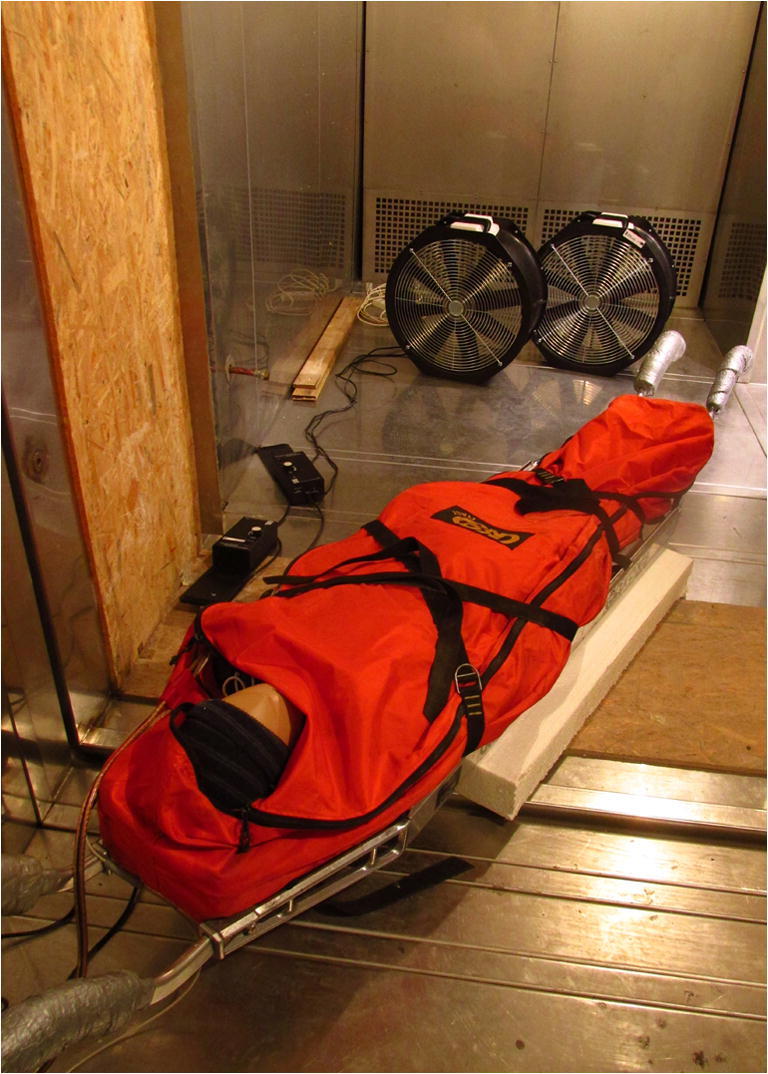
Manikin 1 in the stretcher inside the climatic chamber

Subsequently, we placed the stretcher with Manikin 1 in a climatic chamber in order to measure the heat flux in both weather scenarios (Fig. 1). The stretcher was put on two narrow Styrofoam blocks in order to enable the wind to flow underneath. Each measurement was preceded by a Manikin 1 equilibration period (avg. 60–120 min). After the achievement of thermal equilibrium, the gradient between T_skin_ and T_ambient_ remained constant and the heat flux was recorded.

Active warmers were assessed with Manikin 2, which was dressed in the same clothes as Manikin 1, and placed on the stretcher. We set the same initial temperature of Manikin 2 as the torso temperature of Manikin 1 in the heat loss protocol (26 °C) in order to provide the same temperature gradient between the environment and the “skin.” The temperature was recorded at 1-min intervals. Initially, we measured the heat loss without any warming system in both weather scenarios (A and B), in order to obtain reference values (Q_base_). Subsequently, we tested the warming systems.

Before any measurements were taken, all the heating systems were stored in the climatic chamber for 1 h, excluding the battery of the electrical blanket that was not pre-cooled. Each warming pad was activated and inserted between the shirt and the pullover on the manikin’s chest. We set the electrical system (2) to its maximum temperature of 41 °C. The chemical blanket (3) was opened immediately before starting to take measurements and agitated to allow chemical activation and heat production. We exposed it briefly to the air every 30 min in order to deliver oxygen and maintain the chemical process. Before each measurement was taken, we rewarmed the manikin to 26 °C. All measurements were repeated at least three times. Each time we placed the stretcher at a different angle to the wind direction in order to mimic real conditions. If the differences between the first three results were greater than 10%, an additional measurement was taken, and all results were included in the analysis.

### Statistical analysis

We assessed the normality of data distribution with the Shapiro-Wilk test. Mean values and a standard deviation were used to express normally distributed data. We compared the results of heat gain between the two weather conditions for each warming system, as well as between the different warming systems in the same weather conditions. The values of heat delivered in the 1st, 2nd, and 3rd hour of the experiment were also compared for each warming system separately. Finally, we compared the heat requirements of the virtual patient (the amount of gained heat that would be sufficient to maintain the neutral heat balance) with the amount of heat actually gained from warming systems. Comparisons were made with the use of the Student *t* test for both paired and unpaired data. We used the Statistica version 13 (TIBCO Software Inc.). Statistical significance was defined as *p* < 0.05.

## Results

In three series of measurements with Manikin 2 (two with gel pads and one with the electrical system), one result in each set exceeded a difference of 10% in regard to the others, and an additional measurement was performed. The results of measurements with Manikin 1 did not exceed the difference of 10% in each set.

The thermal resistance of the clothes used in the study was 1.35 clo (0.21 m^2^K/W); that of the stretcher cover was 0.59 clo (0.09 m^2^K/W); and that of the clothes plus stretcher cover was 1.57 clo (0.24 m^2^K/W).

### Heat loss from thermal manikin

The mean heat flux from the skin of Manikin 1 was 62.87 ± 0.12 W/m^2^ in weather A and 108.73 ± 1.97 W/m^2^ in weather B.

### Determination of metabolic heat production

The mean value of M_shiv_ was 139.24 ± 43.97 W/m^2^ while that of M_non-shiv_ was 54.91 ± 4.23 W/m^2^.

### Determination of the total heat balance

The calculated heat storage in the shivering patient model was above zero, namely, 48.53 ± 0.12 W/m^2^ in weather A and 2.66 ± 1.97 W/m^2^ in weather B. In the non-shivering patient model, the heat storage was − 18.94 ± 0.12 W/m^2^ (A) and − 64.81 ± 1.97 W/m^2^ (B), respectively. This means that the non-shivering patient’s heat loss was 122.72 ± 0.75 kJ/h in weather A and 419.94 ± 12.79 kJ/h in weather B.

The total heat balance for the 3.5-h period is given in Table 1.

**Table 1:** Total heat balance of a virtual patient and the heat gain from warming systems during 3.5-h lasting simulated evacuation

Weather conditions	Clinical scenario	Total heat balance (kJ)	Heat gain (kJ)	
A	Shivering	1100.55 ± 2.62	Gel	356.61 ± 55.18
	Non-shivering	− 429.53 ± 2.62	Electrical	274.27 ± 31.86
			Chemical	301.51 ± 35.16
B	Shivering	60.30 ± 44.75	Gel	345.77 ± 45.43
	Non-shivering	− 1469.78 ± 44.75	Electrical	313.27 ± 34.39
			Chemical	318.84 ± 19.66

Weather conditions A: wind chill index of 0 °C; weather conditions B: wind chill index of − 20 °C

### Assessment of heat gain from warming systems

We did not observe any increase in the temperature of Manikin 2 with warming systems applied apart from gel pads in weather A. The highest increase of 0.54 °C occurred 62 min after their application.

The amount of heat gained in 3.5 h was close to 300 kJ and there were no significant differences between both simulated weather conditions for a particular warming system. In weather A, the total heat delivery from the gel warmer was higher than that from the electrical blanket (*p* = 0.04). There were no significant differences in heat delivery between other warming systems in each set of weather conditions. We observed the highest efficacy of gel pads and the electrical system in the first hour of measurement when compared with the 2nd and 3rd hour (*p* < 0.001). Battery exhaustion in the electrical system occurred at an average of 80 min in weather A and 70 min in weather B. The heat gain from the chemical blanket was delayed and started to stabilize within 17 to 25 min after activation.

**Fig. 2:**
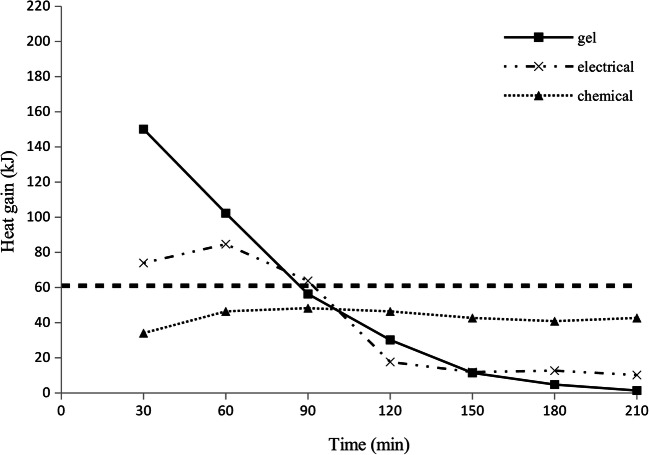
Net heat gain from warming systems calculated for 30-min intervals; the horizontal dotted line represents the heat requirement of a non-shivering patient expressed in kJ/30 min; weather A (wind chill index 0 °C)

**Fig. 3:**
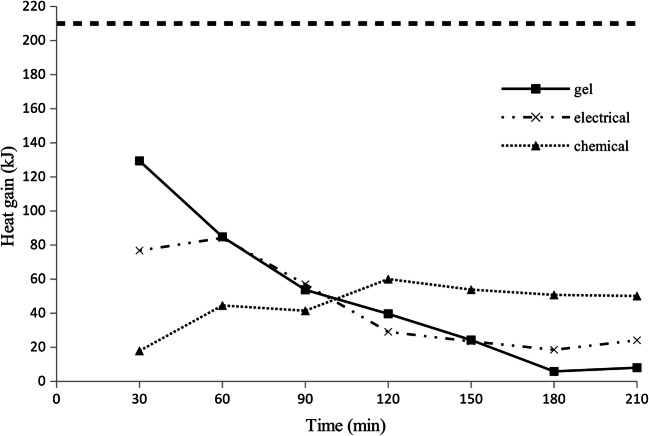
Net heat gain from warming systems calculated for 30-min intervals; the horizontal dotted line represents the heat requirement of a non-shivering patient expressed in kJ/30 min; weather B (wind chill index − 20 °C)

The heat balance of a virtual patient and heat gain from the warming systems are compared in Table 1. In both simulated weather conditions, the heat gain from the warming systems was lower than the heat requirements of the non-shivering victim model, namely, in weather A *p* = 0.07 for gel, *p* < 0.001 for Uniqueresc, and *p* = 0.003 for Ready Heat while in weather B *p* < 0.001 for all systems. The heat delivery to Manikin 2 and the simulated patient’s heat requirements estimated with Manikin 1, calculated for 30-min intervals, are shown in Figs. 2 and 3.

## Discussion

To our knowledge, this is the first study to assess the effectiveness of three warming systems during a mountain rescue operation simulated over 3 h. None of the three tested warming systems was able to compensate for the estimated heat loss in a non-shivering patient in harsh weather conditions. In moderate weather, the tested systems are closer to the patient’s heat requirements, with gel and electrical warmers even being able to exceed these needs in the first 90 min. However, the shivering patient model revealed a positive heat balance that signified the maintenance of normothermia.

Thermal manikins, when properly calibrated, provide an accurate estimation of selected parameters of heat exchange in human beings (Brauer et al. 2002; Psikuta et al. 2016). Indeed, they can be successfully used in cold or dangerous conditions instead of human subjects (Henriksson et al. 2012). In low ambient temperatures, some thermal manikins are not able to maintain target surface temperature due to an insufficient power output (Psikuta et al. 2016). This reduces the reliability of measurements substantially. In our study, the amount of power delivered to each segment was within the performance parameters of the manikin, including the face area where the highest heat flux was observed. Manikins provide standardized conditions without individual variability. However, presetting an appropriate surface temperature is essential for obtaining reliable results concerning heat loss (Wigö and Nilsson 2004). Both a too-high and too-low surface temperature can lead to over- or underestimation of heat loss. Moreover, data concerning skin temperatures in real patients who sustained trauma in a mountainous environment are lacking. Nonetheless, the temperatures we set derived from experimental human studies seem to be adequate. Importantly, Grissom et al. showed that skin temperature, after its initial drop, remains stable for at least 40 min (Grissom et al. 2008). Thus, we may assume that heat loss does not substantially change during the whole period of simulated evacuation.

The total amount of heat delivered by the tested devices was about 300 kJ regardless of the simulated weather conditions. Although this observation confirms the efficacy of all warming systems being tested, its draws one’s attention to the duration and course of their warming capacity. The heat delivery from the warmers was not distributed homogeneously over time. The gel and electrical systems had their highest efficacy in the first hour of the test. The advantage of the chemical blanket over these two systems could be seen in long-lasting rescue operations due to its stability in heat production, despite a delay in the onset of warming. Similar results regarding the gel pads, as well as chemical blankets, were obtained by Dutta et al. (Dutta et al. 2019). The heat delivery/mass ratio of the chemical blanket is the most favorable when compared with the other systems tested in this study. The heating duration of gel and chemical warmers is determined by their construction, whereas the performance of the electrical blanket depends on its battery life. Thus, the efficacy of the electrical system could be improved with backup batteries. The patient’s heat balance that we compared with the efficacy of the warming systems was calculated for an average adult with a body surface area of 1.8 m^2^. In children, due to their smaller body surface, the heat gain from warming systems may be closer to meeting their heat requirements.

Although casualties with preserved shivering are also at risk of post-traumatic hypothermia in cold and windy conditions, it is possible to protect them from cooling with the warming systems tested in this study. It should be noted that we did not use any additional thermal insulation. The reason for such an approach was to create a point of reference for rescuers who plan and prepare equipment for use in rescue operations. Moreover, the great diversity of insulating systems used by mountain rescue teams makes it impossible to test all of them (Podsiadło et al. 2017).

### Practical implications

If the evacuation of a victim lasts for 1 h or less, gel warming pads seem to be a good choice. When an electrical system is used, the battery should be protected from cold during the approach towards the victim (e.g., under a rescuer’s clothing). If possible, backup batteries should be taken in order to allow continuous warming for more than 1 h. Moreover, the chemical blanket should be activated about 20 min before reaching the patient. In addition, mountain rescue teams should be aware of the technical specifications and performance of warming pads.

## Limitations

Although the mean skin temperature and metabolic rates that we assumed were based on studies with human participants, they included healthy volunteers but not trauma victims. Shivering heat production is likely to diminish over time, e.g., due to glycogen resource depletion.

Application of a warming pad can lead to the decrease of an actual patient’s metabolic heat production, a factor that was not included in our calculations due to its unpredictability.

Although the manikins are standardized with little interindividual variability and the advanced manikin functions are based on the mathematical model of human thermodynamics, the measured heat loss should be interpreted with caution and cannot be simply transferred to humans.

The water manikin has no internal temperature gradient (Tc = T_skin_), while the thermal conductivity of copper is much higher than that of the skin, especially when vasoconstriction exists. Thus, the measured heat gain may not reflect precisely the real heat gain in humans.

## Conclusions

From this study, we conclude that a non-shivering (e.g., severely injured) victim of a mountain accident cannot be effectively protected from the onset of hypothermia with a warming system alone. Additional insulation seems to be required for long-duration transport if the patient is exposed to a cold and windy environment. More effective insulation and rewarming methods should be developed for prolonged terrestrial transports of patients with restricted mobility or impaired thermoregulation.

## Electronic supplementary material

ESM 1 (DOCX 27 kb)

## References

[CR1] Brauer A, English MJM, Sander H, Timmermann A, Braun U, Weyland W (2002). Construction and evaluation of a manikin for perioperative heat exchange. Acta Anaesthesiol Scand.

[CR2] Cain JB, Livingstone SD, Nolan RW, Keefe AA (1990). Respiratory heat loss during work at various ambient temperatures. Respir Physiol.

[CR3] Dutta R, Kulkarni K, Steinman AM, Gardiner PF, McDonald GK, Giesbrecht GG (2019). Human responses to 5 heated hypothermia wrap systems in a cold environment. Wilderness Environ Med.

[CR4] Fanger P (1970). Thermal comfort.

[CR5] Gagge AP, Gonzalez RR, Fregly M, Blatteis C (1996). Mechanisms of heat exchange: Biophysics and physiology. Handbook of physiology.

[CR6] Giesbrecht GG, Sessler DI, Mekjavic IB, Schroeder M, Bristow GK (1994). Treatment of mild immersion hypothermia by direct body-to-body contact. J Appl Physiol.

[CR7] Giesbrecht GG, Lockhart TL, Bristow GK, Steinman AM (2005). Thermal effects of dorsal head immersion in cold water on nonshivering humans. J Appl Physiol.

[CR8] Goheen MSL, Ducharme MB, Kenny GP, Johnston CE, Frim J, Bristow GK, Giesbrecht GG (1997). Efficacy of forced-air and inhalation rewarming by using a human model for severe hypothermia. J Appl Physiol.

[CR9] Grant SJ, Dowsett D, Hutchison C, Newell J, Connor T, Grant P, Watt M (2002). A comparison of mountain rescue casualty bags in a cold, windy environment. Wilderness Environ Med.

[CR10] Grissom CK, McAlpine JC, Harmston CH (2008). Hypercapnia effect on core cooling and shivering threshold during snow burial. Aviat Sp Environ Med.

[CR11] Guly HR (1996). Medical aspects of the work of a moorland rescue team. Br J Sports Med.

[CR12] Hamilton RS, Paton BC (1996). The diagnosis and treatment of hypothermia by mountain rescue teams: a survey. Wilderness Environ Med.

[CR13] Hearns S (2003). The Scottish mountain rescue casualty study. Emerg Med J.

[CR14] Henriksson O, Lundgren P, Kuklane K, Holmér I, Naredi P, Bjornstig U (2012). Protection against cold in prehospital care: evaporative heat loss reduction by wet clothing removal or the addition of a vapor barrier—a thermal manikin study. Prehosp Disaster Med.

[CR15] Henriksson O, Lundgren PJ, Kuklane K, Holmér I, Giesbrecht GG, Naredi P, Bjornstig U (2015). Protection against cold in prehospital care: wet clothing removal or addition of a vapor barrier. Wilderness Environ Med.

[CR16] Hultzer MV, Xu X, Marrao C (2005). Pre-hospital torso-warming modalities for severe hypothermia: a comparative study using a human model. Can J Emerg Med.

[CR17] Hurrie DMG, Hildebrand E, Arnould SM, Plett J, Bellan D, Buchel A, Giesbrecht GG (2020). Comparison of electric resistive heating pads and forced-air warming for pre-hospital warming of non-shivering hypothermic subjects. Mil Med.

[CR18] Ingenito EP, Solway J, McFadden ER (1986). Finite difference analysis of respiratory heat transfer. J Appl Physiol.

[CR19] Klauke N, Gräff I, Fleischer A, Boehm O, Guttenthaler V, Baumgarten G, Meybohm P, Wittmann M (2016). Effects of prehospital hypothermia on transfusion requirements and outcomes: a retrospective observatory trial. BMJ Open.

[CR20] Kulkarni K, Hildahl E, Dutta R, Webber SC, Passmore S, McDonald GK, Giesbrecht GG (2019). Efficacy of head and torso rewarming using a human model for severe hypothermia. Wilderness Environ Med.

[CR21] Kumar P, Mcdonald GK, Chitkara R (2015). Comparison of distal limb warming with fluidotherapy and warm water immersion for mild hypothermia rewarming. Wilderness Environ Med.

[CR22] Langhelle A, Lockey D, Harris T, Davies G (2012). Body temperature of trauma patients on admission to hospital: a comparison of anaesthetised and non-anaesthetised patients. Emerg Med J.

[CR23] Layton RP, Mints WH, Annis JF (1983). Calorimetry with heat flux transducers: comparison with a suit calorimeter. J Appl Physiol.

[CR24] Lloyd EL (1975). Airway warming in accidental hypothermia. Br J Sports Med.

[CR25] Lundgren JP, Henriksson O, Pretorius T, Cahill F, Bristow G, Chochinov A, Pretorius A, Bjornstig U, Giesbrecht GG (2009). Field torso-warming modalities: a comparative study using a human model. Prehospital Emerg Care.

[CR26] Lundgren P, Henriksson O, Naredi P, Björnstig U (2011). The effect of active warming in prehospital trauma care during road and air ambulance transportation - a clinical randomized trial. Scand J Trauma Resusc Emerg Med.

[CR27] Martin RS, Kilgo PD, Miller PR, Hoth JJ, Meredith JW, Chang MC (2005). Injury-associated hypothermia: an analysis of the 2004 National Trauma Data Bank. Shock.

[CR28] McLennan JG, Ungersma J (1983). Mountaineering accidents in the Sierra Nevada. Am J Sports Med.

[CR29] Oliver SJ, Brierley JL, Raymond-Barker PC, Dolci A, Walsh NP (2016). Portable prehospital methods to treat near-hypothermic shivering cold casualties. Wilderness Environ Med.

[CR30] Paal P, Gordon L, Strapazzon G, Brodmann Maeder M, Putzer G, Walpoth B, Wanscher M, Brown D, Holzer M, Broessner G, Brugger H (2016). Accidental hypothermia-an update: the content of this review is endorsed by the International Commission for Mountain Emergency Medicine (ICAR MEDCOM). Scand J Trauma Resusc Emerg Med.

[CR31] Peng RY, Bongard FS (1999). Hypothermia in trauma patients. J Am Coll Surg.

[CR32] Podsiadło P, Darocha T, Kosiński S, Sałapa K, Ziętkiewicz M, Sanak T, Turner R, Brugger H (2017). Severe hypothermia management in mountain rescue: a survey study. High Alt Med Biol.

[CR33] Press C, Duffy C, Williams J, Cooper B, Chapman N (2017). Measurements of rates of cooling of a manikin insulated with different mountain rescue casualty bags. Extrem Physiol Med.

[CR34] Pretorius T, Bristow GK, Steinman AM, Giesbrecht GG (2006). Thermal effects of whole head submersion in cold water on nonshivering humans. J Appl Physiol.

[CR35] Pretorius T, Cahill F, Kocay S, Giesbrecht GG (2008). Shivering heat production and core cooling during head-in and head-out immersion in 17 °C water. Aviat Space Environ Med.

[CR36] Psikuta A, Kuklane K, Bogdan A, Havenith G, Annaheim S, Rossi RM (2016). Opportunities and constraints of presently used thermal manikins for thermo-physiological simulation of the human body. Int J Biometeorol.

[CR37] Rauch S, Dal Cappello T, Strapazzon G, Palma M, Bonsante F, Gruber E, Ströhle M, Mair P, Brugger H, International Alpine Trauma Registry Study Group (2018). Pre-hospital times and clinical characteristics of severe trauma patients: a comparison between mountain and urban/suburban areas. Am J Emerg Med.

[CR38] Sanchez-Marin FJ, Calixto-Carrera S, Villaseñor-Mora C (2009). Novel approach to assess the emissivity of the human skin. J Biomed Opt.

[CR39] Shafi S, Elliott AC, Gentilello L (2005). Is hypothermia simply a marker of shock and injury severity or an independent risk factor for mortality in trauma patients? Analysis of a large national trauma registry. J Trauma.

[CR40] Smith LO (2006). Alpine climbing: injuries and illness. Phys Med Rehabil Clin N Am.

[CR41] Sran BJK, McDonald GK, Steinman AM (2014). Comparison of heat donation through the head or torso on mild hypothermia rewarming. Wilderness Environ Med.

[CR42] Thomassen Ø, Færevik H, Østerås Ø, Sunde G, Zakariassen E, Sandsund M, Heltne J, Brattebø G (2011). Comparison of three different prehospital wrapping methods for preventing hypothermia--a crossover study in humans. Scand J Trauma Resusc Emerg Med.

[CR43] Wigö H, Nilsson HO (2004). Application of a thermal manikin to evaluate heat loss rates from people caused by variations in air velocity and air temperature. Int J Vent.

