# Supplementary material for: Efficacy of warming systems in mountain rescue: an experimental manikin study
Source: Int J Biometeorol. 2020 Sep 1;64(12):2161–9. doi: 10.1007/s00484-020-02008-6 (PMC7658064; doi:10.1007/s00484-020-02008-6)
Supplement: Supplementary file 1 — (DOCX 27 kb) [file 484_2020_2008_MOESM1_ESM.docx]

Table 1 (Supplementary Material). Calculation of metabolic heat production in shivering- and non-shivering subjects

| Shivering | | | | | Non-Shivering | | | | |
| --- | --- | --- | --- | --- | --- | --- | --- | --- | --- |
| Reference | Number of subjects | Mean BSA (m^2^) | Mean M (W) | M/BSA (W/m^2^) | Reference | Number of subjects | Mean BSA (m^2^) | Mean M (W) | M/BSA (W/m^2^) |
| (Pretorius et al. 2008) | 8 | 2.1 | 364 | 173.333 | (Hurrie et al. 2020) | 6 | 1.9 | 115 | 60.526 |
| (Hurrie et al. 2020) | 6 | 1.9 | 363 | 191.053 | (Kulkarni et al. 2019) | 6 | 2 | 94 | 47 |
| (Kulkarni et al. 2019) | 6 | 2 | 269 | 134.5 | (Lundgren et al. 2009) | 5 | 2 | 114 | 57 |
| (Lundgren et al. 2009) | 5 | 2 | 195 | 97.5 | (Hultzer et al. 2014) | 6 | 1.9 | 100 | 52.632 |
| (Sran et al. 2014) | 6 | 1.86 | 370.1 | 198.978 | (Pretorius et al. 2006) | 8 | 2.12 | 120 | 56.604 |
| (Kumar et al. 2015) | 7 | 2 | 322 | 161 | (Giesbrecht et al. 2005) | 6 | 2 | 111 | 55.5 |
| (Hultzer et al. 2014) | 6 | 1.9 | 250 | 131.579 |  |  |  |  |  |
| (Thomassen et al. 2011) | 8 | 1.94 | 200 | 103.093 |  |  |  |  |  |
| (Henriksson et al. 2015) | 8 | 1.87 | Not available | 107 |  |  |  |  |  |
| (Giesbrecht et al. 1994) | 6 | 1.93 | 400 | 207.254 |  |  |  |  |  |
| (Grissom et al. 2008) | 11 | 1.92 | 150 | 78.125 |  |  |  |  |  |
| **Total n = 77** | |  | **Mean M (W/m^2^) = 139.24 ±43.97** | | **Total n = 37** | |  | **Mean M (W/m^2^) = 54.91 ±4.23** | |

M – metabolic heat production; BSA – body surface area
